# Improved detection of infection with SARS-CoV-2 Omicron variants of concern in healthcare workers by a second-generation rapid antigen test

**DOI:** 10.1128/spectrum.01768-23

**Published:** 2023-10-13

**Authors:** Jochen M. Wettengel, Katharina Strehle, Catharina von Lucke, Hedwig Roggendorf, Samuel D. Jeske, Catharina Christa, Otto Zelger, Bernhard Haller, Ulrike Protzer, Percy A. Knolle

**Affiliations:** 1 Institute of Virology, School of Medicine and Health, Technical University of Munich (TUM), München, Germany; 2 German Center for Infection Research (DZIF), Munich Partner Site, München, Germany; 3 Institute of Molecular Immunology, School of Medicine and Health, TUM, München, Germany; 4 Coronavirus Diagnostic Center of the University Hospital München Rechts der Isar, School of Medicine, TUM, München, Germany; 5 Institute for AI and Informatics in Medicine Statistics, School of Medicine and Health, TUM, München, Germany; 6 Institute of Virology, Helmholtz Munich, München, Germany; University of Mississippi Medical Center, Jackson, Mississippi, USA

**Keywords:** coronavirus, rapid tests, epidemiology, hospital infections

## Abstract

The continuous genetic evolution of SARS-CoV-2 resulting in the immune escape variant of concern (VoC) Omicron poses a challenge to rapid and accurate diagnosis of infection, especially in high-risk groups such as healthcare workers. We performed a clinical study to determine the diagnostic accuracy and robustness of a second-generation rapid antigen test compared to a first-generation rapid antigen test with an RT-qPCR-based assay as gold standard, for early detection of infections with SARS-CoV-2 Omicron VoC. A total of 428 healthcare workers with COVID-19-associated symptoms or during routine testing participated in the study and completed a questionnaire on infection-associated symptoms, previous SARS-CoV-2 infections, and vaccination status. All participants performed a second- and first-generation rapid antigen test on the day of presentation and repeated the test 2 days later, and a diagnostic SARS-CoV-2 RT-qPCR assay was performed. qPCR-confirmed SARS-CoV-2 infections (*n* = 104) with Omicron VoC (BA.2, BA.4, BA.5) were detected by the first-generation rapid antigen test with a sensitivity of 83.7% (95% CI 75.12%–90.18%), whereas the second-generation rapid antigen test performed with a sensitivity of 89.4% (95% CI 81.9%–94.6%). Increased sensitivity of the second-generation rapid antigen test led to earlier detection of SARS-CoV-2 infection, while lower test sensitivity of the first-generation rapid antigen test was compensated by repeated testing 2 days later. Moreover, direct *in vitro* comparison revealed a lower limit of detection for the second-generation rapid antigen test for isolates of currently circulating Omicron sub-lineages BA.5.2, BQ.1, and XBB.1.

**IMPORTANCE:**

The results from this study demonstrate the usefulness of a second-generation rapid antigen test for early detection of infection with the SARS-CoV-2 Omicron variant of concern (VoC) and reveal a higher sensitivity to detect immune escape Omicron VoCs compared to a first-generation rapid antigen test (89.4% vs 83.7%) in the high-risk group of healthcare workers.

## INTRODUCTION

The SARS-CoV-2 pandemic has led to more than 630 million infections and caused more than 6.6 million COVID-19-associated deaths. The rapid development of COVID-19 vaccines and their introduction into the clinics has helped to significantly reduce morbidity and mortality (1 – 5). The evolution of SARS-CoV-2 and the emergence of new variants of concern (VoCs), however, pose a constant challenge (6, 7). Although being highly efficient in preventing severe COVID-19 disease courses, COVID-19 vaccination does not necessarily establish sterile anti-viral immunity (8). Emerging variants, such as the Omicron VoC and its sub-lineages, carry mutations in their surface proteins enabling increased cellular receptor binding, attributing immune evasion properties, or allowing for infection despite pre-existing SARS-CoV-2-specific antibodies and T cells (9 – 12).

While COVID-19 vaccination provides protection from severe disease after SARS-CoV-2 infection in immune-competent vaccinated individuals, persons at risk because of pre-existing cardiovascular or lung disease, cancer patients, or immune-suppressed patients can still develop severe disease (13). Rapid detection of SARS-CoV-2 infection among healthcare workers is, therefore, important to limit infection transmission to patients at risk. While qPCR-based diagnostics from nasopharyngeal/oropharyngeal swabs is the gold standard for the detection of SARS-CoV-2 infection, readily available and less costly rapid antigen tests allow for self-testing and can help reduce the time to diagnosis (14). Rapid antigen tests have been extensively evaluated for their capacity to detect infections with SARS-CoV-2 VoCs (14 – 26), which have shown a broad variability of their capacity to detect infections at an early time point.

Here, we conducted a diagnostic study to determine the value of a second-generation rapid antigen test regarding improved sensitivity for early detection of Omicron VoCs SARS-CoV-2 infections in healthcare workers in a university hospital setting.

## MATERIALS AND METHODS

### Recruitment of participants

We aimed to compare the sensitivity of a first-generation rapid antigen test to a second-generation rapid antigen test with RT-qPCR-based diagnosis as the gold standard for the early detection of infections with SARS-CoV-2 Omicron sub-lineages in healthcare workers. We initiated a clinical study involving healthcare workers at the University Hospital München Rechts der Isar who reported recent onset of COVID-19-associated symptoms or completed routine diagnostic testing between 24 May and 22 September 2022. The following inclusion criteria were used: participants work at the University Hospital München Rechts der Isar, report a recent onset of COVID-19-associated symptoms or perform routine diagnostic testing, provide written informed consent to participate in this study, and complete all steps of the study (questionnaire, qPCR testing, and two rounds of rapid antigen testing). We excluded participants reporting contraindications for nasal or oropharyngeal swap sampling.

### Rapid antigen tests

Two separate rapid antigen tests [second-generation rapid antigen test—Roche SARS-CoV-2 Rapid Antigen Test 2.0 (9901-NCOV-09G) and first-generation rapid antigen test—SD Biosensor SARS-CoV-2 Rapid Antigen Test Nasal (9901-NCOV-03G)] were performed according to manufacturer’s instructions during the visit of study center by the study team and 2 days later by the study participants themselves. For initial diagnosis, anterior nasal swabs were taken from all participants to perform the two rapid antigen tests, and an oropharyngeal swab was taken for qPCR-based diagnosis of SARS-CoV-2 infection. Two days later, participants repeated antigen testing by themselves. For the repeated antigen tests, participants received training to correctly take anterior nasal swabs and assure comparability of results and were randomized into two groups (order when antigen tests were performed for group 1: second-generation antigen test - > first-generation rapid antigen test, or for group 2: first-generation rapid antigen test - > second-rapid antigen test). The results of the repeated rapid antigen tests were reported by the participants to the study team in documented fashion.

### qPCR-based detection of SARS-CoV-2

Due to better compliance by study participants and no reported loss of sensitivity compared to nasopharyngeal swap samples (27, 28), oropharyngeal swaps (Noble Bioscience) were taken to obtain samples for the determination of viral loads by qPCR. PCR-based detection of SARS-CoV-2 infection was performed in a certified diagnostics laboratory at the Institute of Virology on a Roche Cobas 6800 using the “Cobas SARS-CoV-2 Test Kit,” or on the Qiagen NeuMoDx using the “NeuMoDx SARS-CoV-2 Assay.” Quantification of viral load was achieved by a normalized conversion equation using the ct value determined.

### SARS-CoV-2 genome sequencing

Genome sequencing of SARS-CoV-2 RNA was performed within the Bay-VOC network (29) when viral loads determined by qPCR were above 9.5 × 10^4^ genome equivalents (GE)/mL. RNA was extracted using a Seegene SeePrep32 device, and next-generation sequencing was performed via Illumina next-generation sequencing to determine the SARS-CoV-2 Pango lineage.

### Performance of rapid antigen tests *in vitro*

Clinical isolates of SARS-CoV-2 Omicron swap samples (BA.5.2.3, BQ.1 and XBB.1, confirmed by viral genome sequencing) were serially diluted in viral transport media. Subsequently, antigen test extraction buffer tubes were filled with 100 µL of the dilutions and vortexed. First- and second-generation rapid antigens tests were performed according to the manufacturer’s instructions.

### Statistical analyses

Statistical analysis was performed using GraphPad Prism (GraphPad Software, San Diego, CA, USA) and R (R Foundation for Statistical Computing, Vienna, Austria). Means and standard deviations are presented for quantitative data, absolute and relative frequencies for categorical data. For calculation of sensitivity, specificity, positive predictive value, and negative predictive value of the two rapid antigen tests, results of the qPCR tests were considered as gold standard. Exact 95% confidence intervals (CIs) were estimated and are presented (Clopper-Pearson intervals). A logistic regression model was fit to the data to estimate the detection probability of both rapid antigen tests in dependence of the viral load using data from individuals with positive qPCR tests. Results of the antigen tests were used as dependent variable (positive = 1, negative = 0) and the log-viral load as independent variable.

## RESULTS

**Fig 1:**
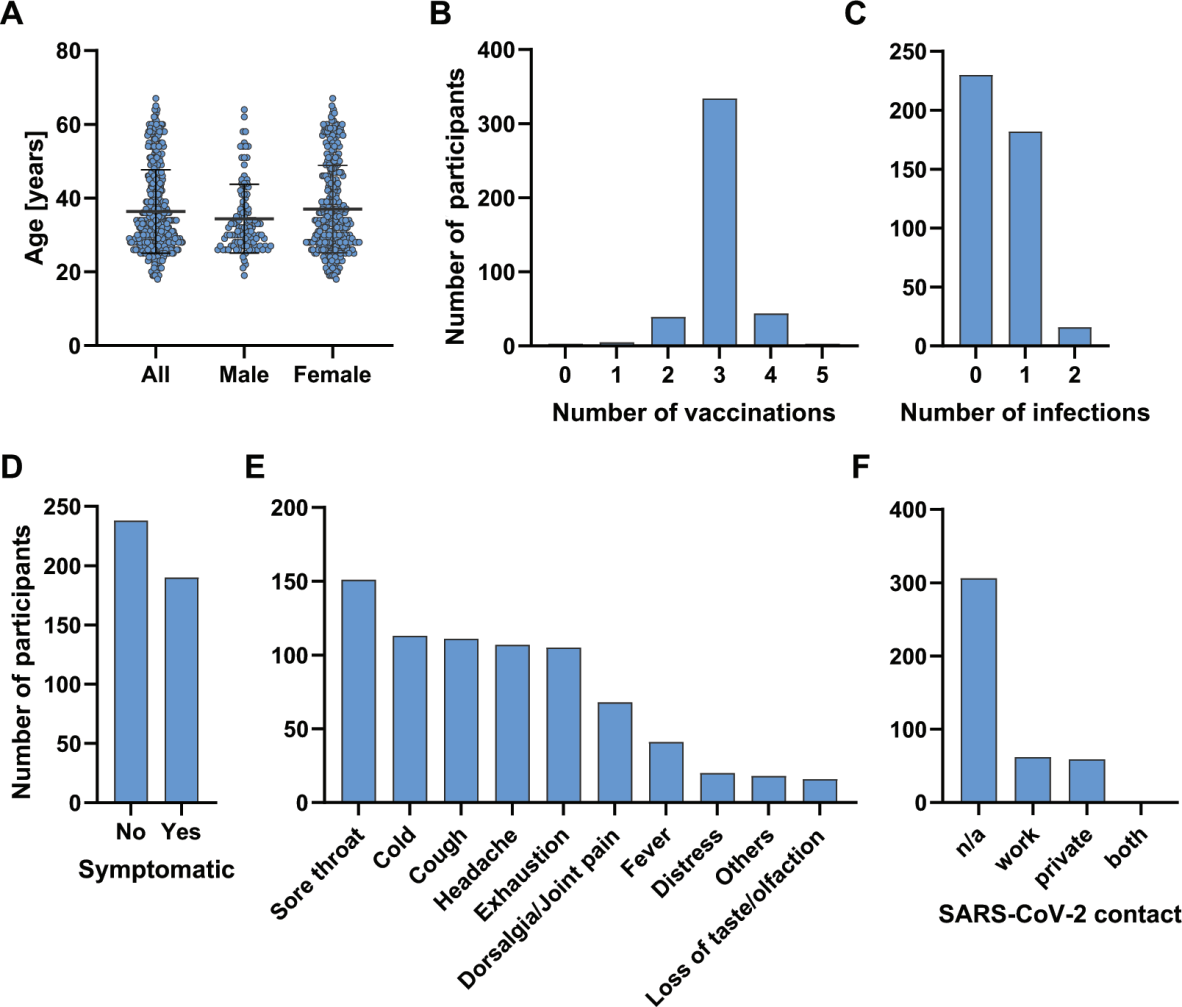
Characteristics of study participants. (**A**) Age of participants enrolled into the study, bars indicate mean and standard deviation. (**B**) Numbers of COVID-19 vaccinations received, or (**C**) numbers of prior SARS-CoV-2 infections. (**D**) Total numbers of participants reporting COVID-19-associated symptoms within 3 days prior inclusion into the study. (**E**) Number of participants reporting specific symptoms and (**F**) reporting risk contacts with SARS-CoV-2-infected persons. The number of study participants with no knowningly contact with a SARS-CoV-2 infected person is indicated as n/a.

From 436 healthcare workers reporting at the Coronavirus Diagnostic Center of the University Hospital München Rechts der Isar with COVID-19-associated symptoms or due to routine testing for SARS-CoV-2 infection in the absence of symptoms (Table 1), we included 428 participants into the study in two randomized study groups. The mean age of study participants was 36.3 years (range 18–67 years) with a similar age range in female (mean 34.4 years; range 18–67 years) and male (mean 37.0 years; range 19–64 years) participants (Fig. 1A; Table 1). All but eight participants (98.1%) had received at least two COVID-19 mRNA vaccinations and 198 participants (46.3%) reported one or two SARS-CoV-2 infections in the past (Fig. 1B and C; Table 1). And 190 participants (44.4%) reported recent onset of COVID-19-associated symptoms, mostly sore throat, whereas 238 participants (55.6%) were asymptomatic (Fig. 1D and E; Table 1). While 306 participants (71. 5%) did not report or recapitulate a contact to a SARS-CoV-2-positive person in the past 2 days, there were 59 participants (13.8%) with private and 62 participants (14.5%) with work-related contacts (Fig. 1F).

**TABLE 1:** Characteristics of study participants

Characteristics	Participants (*n* = 428)
Sex	
Female	313
Male	115
Age	
All mean (SD)	36.33 (11.29)
Female mean (SD)	34.41 (9.33)
Male mean (SD)	37.04 (11.87)
SARS-CoV-2 vaccination, doses	
#0	3
#1	5
#2	39
#3	334
#4	44
#5	3
Previous SARS-CoV-2 infections	
#0	230
#1	182
#2	16
Presenting with COVID-19 typical symptoms	
No	238
Yes	190
Symptoms	
Fever	41
Headache	107
Cough	111
Sore throat	151
Distress	20
Cold	113
Loss of taste/olfaction	16
Dorsalgia/joint pain	68
Exhaustion	105
Others	18
Contact with a SARS-CoV-2-infected person	
Private	59
Work	62
Both	1
n/a^*a*^	306

^*a*^ n/a, The number of study participants with no known contact with a SARS-CoV-2-infected person is indicated as n/a

**Fig 2:**
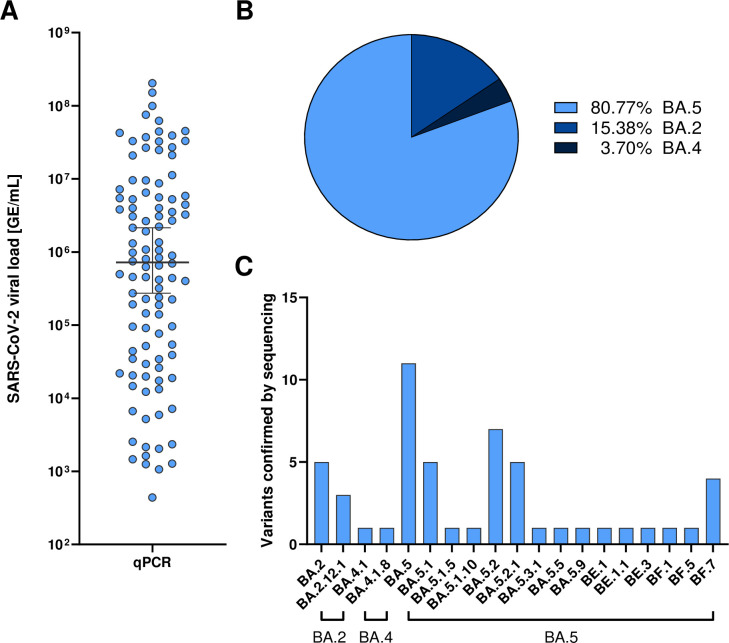
Quantitative results of SARS-CoV-2 RNA levels by qPCR and results from viral genome sequencing. (**A**) Quantitative qPCR results of SARS-CoV-2 RNA as genome equivalents/mL detected in oropharyngeal swabs from study participants. (**B**) Frequencies of Omicron VoC sub-lineages BA.5, BA.2, and BA.4 detected by full viral genome sequencing of samples with ≥9.5 × 10^4^ GE/mL. (**C**) Numbers of infections with different SARS-CoV-2 Omicron Pango lineages.

In 104 out of 428 participants (24.3%), we detected SARS-CoV-2 RNA by qPCR from swab samples and determined viral loads (Fig. 2A). Results from SARS-CoV-2 qPCR ranged from 4.4 × 10^2^ GE/mL to 1.4 × 10^9^ GE/mL with a mean of 2.4 × 10^7^ GE/mL. SARS-CoV-2 sub-lineage analysis of 52/70 samples with a viral load ≥9.5 × 10^4^ GE/mL by full viral genome sequencing revealed a majority of infections with Omicron BA.5 followed by BA.2 and BA.4 (Fig. 2B and C). SARS-CoV-2 genomes not successfully sequenced (18/70; 25.7%) due to insufficient sample material are indicated as n/a. The occurrence of SARS-CoV-2 Omicron Pango lineages over the time of this study in study participants was consistent with their occurrence in the population across Bavaria, Germany (29) (Table S1).

Compared to qPCR-based detection of SARS-CoV-2 RNA as gold standard, we detected 93 out of 104 infections with the second-generation rapid antigen test (Table 2) with 11 false-negative results (Table S2) and 2 false-positive results from testing of 324 qPCR-negative participants (Fig. 2A). Overall, this resulted in a sensitivity of 89.4% (93/104), a specificity of 99.4% (322/324), a positive predictive value of 97.9% (93/95), and a negative predictive value of 96.7% (322/333) (Table 2) for the second-generation rapid antigen test.

**TABLE 2:** Performance characteristics of the two rapid antigen tests

qPCR	First-generation rapid antigen test			Second-generation rapid antigen test		
	Positive	Negative	Total	Positive	Negative	Total
Positive	87	17	104	93	11	104
Negative	2	322	324	2	322	324
Total			428			428
Sensitivity (95% CI)	83.65% (75.12%–90.18%)			89.42% (81.86%–94.60%)		
Specificity (95% CI)	99.38% (97.79%–99.93%)			99.38% (97.79%–99.93%)		
Positive predictive value (95% CI)	97.75% (92.12%–99.73%)			97.89% (92.60%–99.74%)		
Negative predictive value (95% CI)	94.99% (92.09%–97.05%)			96.70% (94.17%–98.34%)		

The first-generation rapid antigen test detected 87 out of 104 SARS-CoV-2 infections, with 17 false-negative results (Table S2) and 2 false-positive results (Table 2). This resulted in a sensitivity of 83.6% (87/104), a specificity of 99.4% (322/324), a positive predictive value of 97.8% (87/89), and a negative predictive value of 95% (322/339) (Table 2).

**Fig 3:**
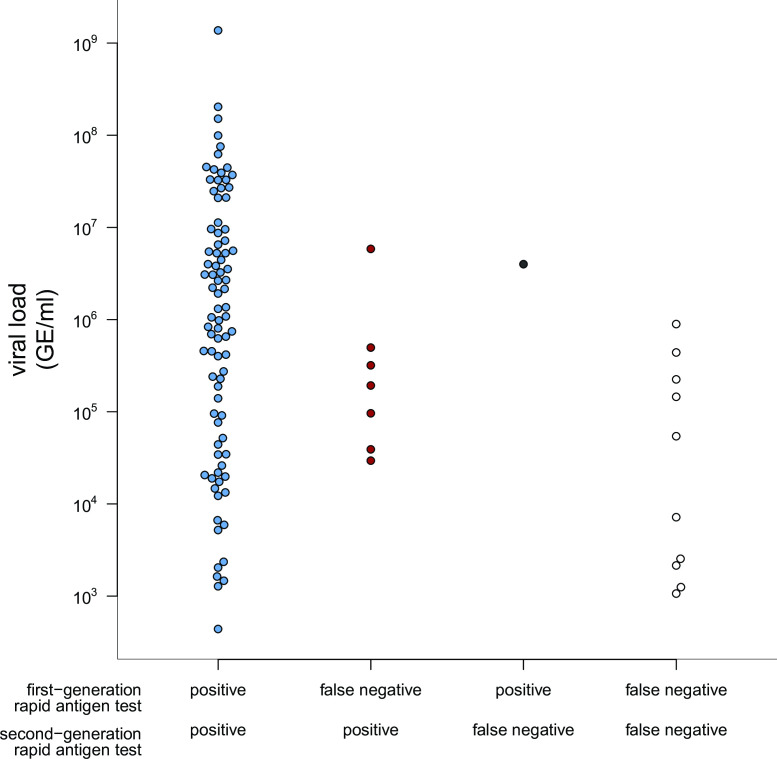
SARS-CoV-2 RNA levels related to results obtained by first- or second-generation rapid antigen tests. Quantitative SARS-CoV-2 RNA levels from participants with both positive first- and second-generation rapid antigen tests (blue), only positive second-generation rapid antigen test (red) or only positive first-generation rapid antigen test (black), or false negative tests in both tests (open circles).

**Fig 4:**
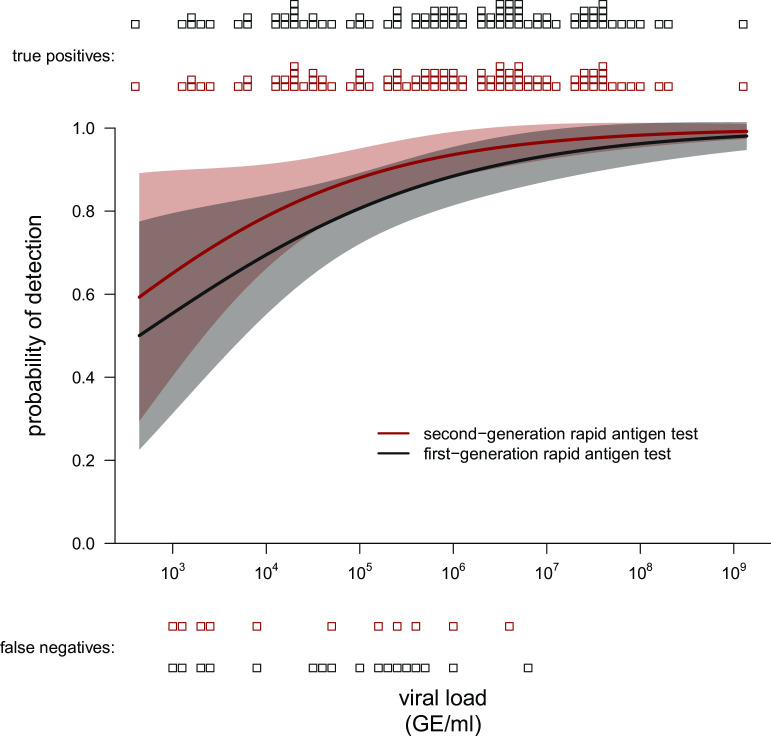
Detection probability for first- and second-generation rapid antigen tests. Detection probability by first-generation and second-generation rapid antigen test stratified according to different SARS-CoV-2 RNA levels quantified by qPCR.

Side-by-side comparison of false-negative results from rapid antigen tests with qPCR results revealed that neither of rapid antigen tests had a clear viral RNA cut-off level for detection of infection. While both tests failed to detect infections of some individuals with low RNA levels of SARS-CoV-2, each test failed to detect an individual sample with RNA levels >10^6^ GE/mL (Fig. 3). Correlating the rapid antigen test results with SARS-CoV-2 RNA levels revealed a higher detection probability for infection by the second-generation rapid antigen tests, particularly for infections with SARS-CoV-2 viral loads between 10^4^ and 10^6^ GE/mL (Fig. 4). Of note, seven patients without reporting any symptoms were SARS-CoV-2 RNA qPCR positive, six of whom were also detected by both rapid antigen tests.

Both rapid antigen tests were repeated 48 hours later by participants yielding the following five observations:

1.) In seven qPCR-positive participants (SARS-CoV-2 RNA levels ranging from 2.9 × 10^3^ to 5.8 × 10^6^ GE/mL) with a positive second-generation rapid antigen test only on the first time point, both first- and second-generation rapid antigen tests were positive on the second time point of analysis (Table 3).

**TABLE 3:** Results from the two time points of rapid antigen testing (day 1, day 3)

First-generation rapid antigen test (day 1)	Second-generation rapid antigen test (day 1)	qPCR (day 1)	First-generation rapid antigen test (day 3)	Second-generation rapid antigen test (day 3)	Viral load (GE/mL)	Pango lineage	Prior SARS-CoV-2 infections	Vaccinations
Positive	Negative	Positive	Positive	Positive	3.99E+06	BA.5.2	0	3
Negative	Positive	Positive	Positive	Positive	2.95E+04	n/a^*a*^	0	3
Negative	Positive	Positive	Positive	Positive	3.91E+04	n/a	0	4
Negative	Positive	Positive	Positive	Positive	9.62E+04	n/a	0	3
Negative	Positive	Positive	Positive	Positive	1.93E+05	n/a	0	3
Negative	Positive	Positive	Positive	Positive	3.19E+05	BA.2.12.1	0	3
Negative	Positive	Positive	Positive	Positive	4.97E+05	BA.2.12.1	0	3
Negative	Positive	Positive	Positive	Positive	5.85E+06	BE.1	0	3
Negative	Negative	Positive	Positive	Positive	7.17E+03	n/a	0	3
Negative	Negative	Positive	Positive	Positive	5.43E+04	n/a	0	4
Negative	Negative	Positive	Positive	Positive	1.45E+05	BF.7	0	3
Negative	Negative	Positive	Positive	Positive	4.39E+05	BA.5.2.1	0	4
Negative	Negative	Positive	Positive	Positive	8.94E+05	n/a	0	3
Negative	Negative	Positive	Negative	Negative	1.06E+03	n/a	1	3
Negative	Negative	Positive	Negative	Negative	1.25E+03	n/a	1	3
Negative	Negative	Positive	Negative	Negative	2.15E+03	n/a	0	3
Negative	Negative	Positive	Negative	Negative	2.54E+03	n/a	0	3
Negative	Negative	Positive	Negative	Negative	2.24E+05	n/a	0	3
Positive	Positive	Positive	Negative	Negative	4.39E+02	n/a	0	3
Positive	Positive	Positive	Negative	Negative	5.93E+03	n/a	0	3
Negative	Negative	Negative	Positive	Positive	Negative	n/a	0	2
Positive	Positive	Negative	Positive	Positive	Negative	n/a	0	3
Positive	Positive	Negative	Negative	Negative	Negative	n/a	0	3

^*a*^ SARS-CoV-2 genomes with a viral load above 9.5 × 10^4 GE/mL, which could not successfully be sequenced, or with a viral load below 9.5 × 10^4 GE/mL, which were not sequenced are indicated as n/a.

2.) In five qPCR-positive participants (viral RNA levels ranging from 7 × 10^3^ to 8.9 × 10^5^ GE/mL), both rapid antigen tests failed to detect infection at the first time point, but both rapid antigen tests gave positive results when performed 2 days later (Table 3). These two observations are most compatible with early stages of SARS-CoV-2 infection, where during the following course of infection, increased levels of viral nucleocapsids from infected and dying epithelial cells facilitate detection by rapid antigen tests and indicate a higher detection sensitivity of the second-generation rapid antigen test compared to the first-generation rapid antigen test.

3.) In four participants with very low viral RNA levels (1–2 × 10^3^ GE/mL) and in one participant with 2.2 × 10^5^ GE/mL, repetition of the two rapid antigen tests 2 days later also failed to detect infection (Table 3), highlighting the previously described limitations of rapid antigen tests for the detection of low viral load infections compared to qPCR-based testing.

4.) In two individuals with low viral RNA levels (4.3 × 10^2^ and 5.9 × 10^3^ GE/mL), both rapid antigen tests were positive during the initial test but showed negative results on the second time of testing (Table 3), most likely compatible with clearance of infection at the second time point of testing.

5.) Three false-positive results from qPCR-negative study participants were obtained with two false-positive results obtained by each rapid antigen test (Table 3).

Combining results from the two time points of analysis, both rapid antigen tests detected 99 out of 104 SARS-CoV-2-infected qPCR-positive participants (overall sensitivity of 95.2%) with no significant differences in the two randomized groups, prior SARS-CoV-2 infections, or number of vaccinations (Table S3).

**Fig 5:**
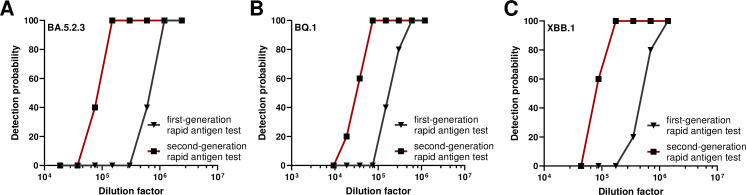
Detection limits for different SARS-CoV-2 Omicron VoCs for first- and second-generation rapid antigen tests. Serial dilution of SARS-CoV-2 VoC clinical isolates: (**A**) BA.5.2.3, (**B**) BQ1, and (**C**) XBB1 and detection by first- and second-generation rapid antigen tests.

While our study did not include participants with infections by current circulating SARS-CoV-2 Omicron sub-lineages, such as BA.5.2.3, BQ.1, and XBB.1, emerging after the end of the study, we evaluated the first- and second-generation rapid antigen tests for their capacity to detect SARS-CoV-2 nucleocapsid antigens from clinical isolates of these VoC sub-lineages. Side-by-side comparison of the two rapid antigen tests using serial dilution of these defined clinical isolates demonstrated that both rapid antigen tests recognized nucleocapsids from BA.5.2.3, BQ.1, and XBB.1 (Fig. 5). In accordance with our previous data, the second-generation rapid antigen test was capable to detect approximately eightfold higher dilutions of the clinical isolates of SARS-CoV-2 omicron VoCs compared to the first-generation rapid antigen test (Fig. 5), indicating also a higher sensitivity to detect the current circulating SARS-CoV-2 omicron VoC sub-lineages.

## DISCUSSION

SARS-CoV-2 continuously evolved over the last 3 years leading to emergence of VoCs that, through mutations in viral surface genes, can escape immune responses generated from previous infections (30). Early detection of SARS-CoV-2 infection, in particular infections with novel mutated SARS-CoV-2 VoCs, is therefore paramount to prevent infection of persons at risk such as hospitalized and severely ill patients. Beyond the more time-consuming and more expensive qPCR-based detection of SARS-CoV-2 RNA, rapid antigen tests can contribute to such early detection of SARS-CoV-2 infection in hospital employees in order to prevent spread of infection to vulnerable patient populations (31, 32). We have systematically explored the capacity of a first- and second-generation rapid antigen test for early detection of SARS-CoV-2 among hospital workers. While other studies have demonstrated the ability of different rapid antigen tests to detect infections with newly emerging Omicron sub-lineages (14, 33, 34), this study for the first time reports on how a higher capacity for the detection of viral nucleocapsids from emerging SARS-CoV-2 Omicron VoCs translates into improved detection of infection with these VoCs in a high-risk cohort of healthcare workers.

Comparing the two antigen rapid tests side by side and at two consecutive time points, we observed in 7 out of 104 infected individuals that a second-generation rapid antigen test detected infections with SARS-CoV-2 omicron VoCs earlier, which points toward a higher sensitivity to detect viral nucleocapsids early in infection. The sensitivity of the second-generation rapid antigen test to detect SARS-CoV-2 infection with immune escape VoCs, evaluated in this study under real-life conditions, was better compared to previously published results for other tests (35, 36) and met the 75% sensitivity required by regulatory agencies in Germany. However, no clear quantitative cut-off level could be defined for SARS-CoV-2 genome equivalents, above which detection of infection was achieved by the two rapid antigen tests evaluated here. These results suggest that other parameters were more relevant for detection of infection through rapid antigen tests such as the extent of death of SARS-CoV-2-infected cells in the upper respiratory tract and the associated release of viral nucleocapsids relevant for detection by rapid antigen tests, both factors that do not necessarily correlate with viral RNA levels detected by qPCR.

Due to its high sensitivity and specificity (37, 38), the results of the diagnostic RT-qPCRs were considered to be the gold standard for the detection of SARS-CoV-2 infection in this study. In three cases, the qPCR result was negative, and the results of both rapid antigen tests were positive on day 1, day 3 or day 1, and day 3. While this indicates false-positive rapid antigen test results, concurrent positive results from two rapid antigen tests may also point toward a false-negative qPCR result caused by erroneous swap sampling, handling, or processing.

Overall, the results from this study will help to refine strategies for early detection of SARS-CoV-2 infection with emerging VoCs like Omicron in symptomatic but also in asymptomatic healthcare workers contributing to the prevention of the spread of SARS-CoV-2 infection to vulnerable patient populations in hospitals.

## SUPPLEMENTAL MATERIAL

Supplemental file 1 spectrum.01768-23-s0001.docx Table S1.

Supplemental file 2 spectrum.01768-23-s0002.docx Table S2.

Supplemental file 3 spectrum.01768-23-s0003.xlsx Table S3.

Supplemental file 4 spectrum.01768-23-s0004.docx Table S3 description.

ASM does not own the copyrights to Supplemental Material that may be linked to, or accessed through, an article. The authors have granted ASM a non-exclusive, world-wide license to publish the Supplemental Material files. Please contact the corresponding author directly for reuse.
