# Supplementary material for: Improved detection of infection with SARS-CoV-2 Omicron variants of concern in healthcare workers by a second-generation rapid antigen test
Source: Microbiol Spectr. 2023 Oct 13;11(6):e01768-23. doi: 10.1128/spectrum.01768-23 (PMC10714798; doi:10.1128/spectrum.01768-23)
Supplement: Supplemental file 1 — Table S1. [file spectrum.01768-23-s0001.docx]

**Supplementary Table 1| Pango lineages identified during the study compared to the percentage circulating in Bavaria.**

|  | **Percentage circulating in Bavaria** | | | **Study week** | **RaCoMRI study** | | |
| --- | --- | --- | --- | --- | --- | --- | --- |
| **Calender week** | BA.2 | BA.4 | BA.5 |  | BA.2 | BA.4 | BA.5 |
| 16/22 | 91% | 0% | 0% | --- | --- | --- | --- |
| 17/22 | 53% | 0% | 3% | --- | --- | --- | --- |
| 18/22 | 96% | 0% | 1% | --- | --- | --- | --- |
| 19/22 | 96% | 0% | 2% | --- | --- | --- | --- |
| 20/22 | 96% | 0% | 2% | --- | --- | --- | --- |
| 21/22 | 95% | 1% | 4% | 1 | 1 | --- | --- |
| 22/22 | 93% | 1% | 5% | 2 | 2 | --- | 3 |
| 23/22 | 88% | 1% | 10% | 3 | 4 | --- | 3 |
| 24/22 | 29% | 9% | 61% | 4 | 1 | --- | 5 |
| 25/22 | 63% | 4% | 32% | 5 | --- | --- | --- |
| 26/22 | 38% | 7% | 55% | 6 | --- | --- | 1 |
| 27/22 | 28% | 6% | 66% | 7 | --- | --- | 3 |
| 28/22 | 57% | 18% | 23% | 8 | --- | --- | --- |
| 29/22 | 40% | 28% | 31% | 9 | --- | --- | 3 |
| 30/22 | 6% | 7% | 86% | 10 | --- | --- | 1 |
| 31/22 | 5% | 5% | 89% | 11 | --- | 1 | 1 |
| 32/22 | 3% | 5% | 91% | 12 | --- | --- | 4 |
| 33/22 | 2% | 0% | 96% | 13 | --- | --- | 5 |
| 34/22 | 2% | 5% | 91% | 14 | --- | --- | 2 |
| 35/22 | 0% | 3% | 95% | 15 | --- | --- | 4 |
| 36/22 | 1% | 3% | 94% | 16 | --- | --- | 1 |
| 37/22 | 1% | 2% | 92% | 17 | --- | --- | 3 |
| 38/22 | 1% | 3% | 91% | 18 | --- | 1 | 3 |
| 39/22 | 0% | 3% | 92% | --- | --- | --- | --- |
| 40/22 | 0% | 4% | 94% | --- | --- | --- | --- |
| 41/22 | 0% | 3% | 90% | --- | --- | --- | --- |
| 42/22 | 1% | 3% | 83% | --- | --- | --- | --- |
| 43/22 | 1% | 1% | 79% | --- | --- | --- | --- |
