# Supplementary material for: Improved detection of infection with SARS-CoV-2 Omicron variants of concern in healthcare workers by a second-generation rapid antigen test
Source: Microbiol Spectr. 2023 Oct 13;11(6):e01768-23. doi: 10.1128/spectrum.01768-23 (PMC10714798; doi:10.1128/spectrum.01768-23)
Supplement: Supplemental file 2 — Table S2. [file spectrum.01768-23-s0002.docx]

**Table 1 | Characteristics of study participants**

| **Characteristics** | **Participants (n = 428)** |
| --- | --- |
| Sex | |
| Female | 313 |
| Male | 115 |
| Age | |
| All Mean (SD) | 36.33 (11.29) |
| Female Mean (SD) | 34.41 (9.33) |
| Male Mean (SD) | 37.04 (11.87) |
| SARS-CoV-2 vaccination, doses | |
| #0 | 3 |
| #1 | 5 |
| #2 | 39 |
| #3 | 334 |
| #4 | 44 |
| #5 | 3 |
| Previous SARS-CoV-2 infections | |
| #0 | 230 |
| #1 | 182 |
| #2 | 16 |
| Presenting with COVID-19 typical symptoms | |
| No | 238 |
| Yes | 190 |
| Symptoms | |
| Fever | 41 |
| Headache | 107 |
| Cough | 111 |
| Sore throat | 151 |
| Distress | 20 |
| Cold | 113 |
| Loss of taste/olfaction | 16 |
| Dorsalgia/joint pain | 68 |
| Exhaustion | 105 |
| Others | 18 |
| Contact with a SARS-CoV-2 infected person | |
| private | 59 |
| work | 62 |
| both | 1 |
| n/a | 306 |
