# Supplementary material for: Improved detection of infection with SARS-CoV-2 Omicron variants of concern in healthcare workers by a second-generation rapid antigen test
Source: Microbiol Spectr. 2023 Oct 13;11(6):e01768-23. doi: 10.1128/spectrum.01768-23 (PMC10714798; doi:10.1128/spectrum.01768-23)
Supplement: Supplemental file 4 — Table S3 description. [file spectrum.01768-23-s0004.docx]

**Supplementary Table 3 | All data collected in this study**

*See Excel Sheet*
